# How frequent is osteogenesis imperfecta in patients with idiopathic osteoporosis?: Case reports: Erratum

**DOI:** 10.1097/MD.0000000000008141

**Published:** 2017-09-15

In the article, “How frequent is osteogenesis imperfecta in patients with idiopathic osteoporosis?: Case reports”,^[1]^ which appeared in Volume 96, Issue 35 of *Medicine*, Dr. Christian Windpassinger's affiliation should have appeared as “Institute of Human Genetics,

Medical University of Graz, Graz, Austria.”
